# The Use of Five Public Health Themes in Understanding the Roles of Misinformation and Education Toward Disparities in Racial and Ethnic Distribution of COVID-19

**DOI:** 10.7759/cureus.30008

**Published:** 2022-10-06

**Authors:** Olumide M Arigbede, Olabimpe B Aladeniyi, Sarah G Buxbaum, Oluwatomilola J Arigbede

**Affiliations:** 1 Institute of Public Health, College of Pharmacy and Pharmaceutical Sciences, Florida Agricultural and Mechanical University, Tallahassee, USA; 2 School of Sciences, Department of Statistics, Federal University of Akure, Akure, NGA; 3 Division of Healthcare Professions, Tallahassee Community College, Tallahassee, USA

**Keywords:** covid-19, epidemiologic model, public health themes, health literacy, public health policies, health disparities, underserved populations, evidence-based public heath, infodemic, misinformation

## Abstract

The distribution of coronavirus disease 2019 (COVID-19) infection across the historically marginalized populations in the United States (US) has consistently been inequitable. In addition, systemic racism and prejudice, which have existed for decades, have caused a lack of faith in public health and medical experts and have resulted in the epidemic of misinformation. To counteract the COVID-19 pandemic and widespread misinformation, the political establishment and public health experts must work collaboratively. And because they are closely associated, there had been a significant increase in the prevalence of the disease as well as a spike in the number of hospitalizations and fatalities. Public health professionals have investigated a number of epidemiological strategies to stop the spread of the virus and mitigate its effects, but false information released via various media sources has caused serious harm to a number of people. To create the framework and guidelines for protecting audiences from lies and deceit, and eradicating false information before taking root in society, it is essential to understand the types of misinformation that are being spread since the disadvantaged and uneducated communities suffer disproportionately as a result. According to studies, spreading false information could have a negative impact on a country’s health outcomes, as well as its economic and social well-being, if not immediately refuted. Public health themes, such as evidence-based programs, health communication, and health policy, among others need to be evaluated and put into action in order to prevent the dissemination of incorrect information. This review examines a number of public health themes, such as policy and evidence-based strategies that might help in the fight against misinformation that has wreaked havoc on families and communities, particularly the underserved and uninformed populations.

## Introduction and background

Background

On 12 March 2020, the World Health Organization (WHO) declared coronavirus disease a pandemic [1]. Coronavirus disease 2019 (COVID-19), a respiratory disease caused by the novel coronavirus known as severe acute respiratory syndrome coronavirus 2 (SARS-CoV-2), has spread worldwide, affecting nearly all territories and countries. By mid-2020, coronavirus had affected about 25 countries, including the US, Japan, Germany, Singapore, South Korea, and a few African countries, but by the end of August 2022, it had spread to about 228 countries and territories around the world [2,3]. To date, COVID-19 has had a substantial adverse impact on human lives, the economy, and increased poverty globally, disproportionately affecting those in underserved communities. The coronavirus disease outbreak was first reported in Wuhan, China [4]. WHO cautioned countries to take precautionary measures, also known as the stringency index, to curb the spread of the disease. These measures included lockdowns, restriction of population movements, restrictions on gathering, physical distancing, wearing face masks, and washing hands. Notably, COVID-19 affected not only the health of populations but also every other aspect of their lives. In addition to the dramatic loss of life, COVID-19 undermined food systems and job security, exposing millions of people to potential extreme poverty. More importantly, later studies have demonstrated the disparate impact of COVID-19 on populations. Minority populations, especially African Americans and Hispanics, have been disproportionately affected by COVID-19 in terms of lost lives, loss of jobs, and increased poverty; levels of education and misinformation have been shown to fuel these disparities [5,6].

Multiple factors put people at higher risk of contracting COVID-19 and dying from the disease, thereby, creating disparities. Some of these factors include age, education attained, nature of the job, co-morbidity, race/ethnicity, and gender. According to COVID-19 data, older individuals and/or individuals with conditions such as diabetes, obesity, chronic kidney disease, and cardiovascular disease are at a higher risk of infection from COVID-19. In the United States, people of certain races/ethnicities, especially African Americans and Hispanics, were more affected by COVID-19 compared to any other race/ethnicity [7-9]. The precipitating mechanism for the higher prevalence of COVID-19 among African Americans and Hispanics is attributed to multiple factors. These precipitating mechanisms include accumulating co-morbidities and socioeconomic structures [7]. Notably, socioeconomic structures contribute to a higher prevalence of COVID-19 among these populations due to low education levels and misinformation triggered by some driving factors like work conditions (some frontline workers share false facts) and residential status (such as multi-family housing). Socioeconomic structures in the US drive low educational levels, especially around the susceptible fraction of the population, and have higher chances of misinformation [10]. Some of these issues can be resolved by implementing an evidence-based public health (EBPH) strategy that educates competent public health professionals on how to successfully implement research-based interventions at the community level to enhance health outcomes [11]. The aim of this review is to evaluate how levels of education and misinformation interacted to cause a disparate distribution of COVID-19 among susceptible populations in the US. In the review, five public-health themes which include evidence-based programs, public-health leadership, communication and advocacy, vulnerable populations and health disparities, and policy and evaluation would be incorporated.

Problem statement

Numerous studies have demonstrated how COVID-19 disproportionately affected ethnic minorities in the United States and other developed countries. In mid-2020, over 2.4 million COVID-19 cases had been confirmed in the US, and 120,000 confirmed causalities [8], and by August 2022, the country had recorded over 92.9 million and over 1.04 million confirmed and death cases, respectively [2,12]. This shows that the statistics has jumped significantly with over 250% increase in incidence and over 5,000% increase in mortality rates, respectively [13]. However, the reported COVID-19-related death rate was 2.5 times higher among African Americans compared to white non-Hispanic populations [8]. According to data from the American Public Media Research Lab, African Americans comprised 24% of all deaths linked to COVID-19 despite making up only 12% of the US Population [8]. Additionally, considering their low levels of education, a recent study by Pathak et al. [14] found that African Americans and Hispanics had 2.6 and 3.1 times the risk of dying from COVID-19, respectively, compared to white non-Hispanics. Therefore, it is evident that COVID-19 case distribution varies by race/ethnicity and the educational level attained, with African Americans and Hispanics disproportionately affected. The rate of COVID-19 deaths has been shown to be higher among African Americans and Hispanics at 88 per 100,000 and 54 per 100,000, respectively [7]. When compared to white non-Hispanic Americans, African Americans and Hispanics are 3.6 and 3.2 times more likely to die from COVID-19 [7]. These disparities even extend when stratified by age groups. For example, in the less age category where COVID-19 deaths are low; African Americans and Hispanics still exhibit higher death rates compared to white non-Hispanic Americans [7]. Similarly, and according to Feldman and Bassett [5], there would have been a considerable decrease (not less than 48 percent) in COVID-19 mortality if all groups had experienced the same death rates stratifying by age categories and using college-educated non-Hispanic white people as the referent.

Consequently, African Americans and Hispanics have been disproportionately affected by COVID-19 not only in terms of disease prevalence and mortality but also socio-economically. The socioeconomic status of African Americans and Hispanics is a major factor that explains the disproportionate impact of COVID-19. Factors such as education level and occupation interact to create a perfect environment for the virus to thrive. For instance, due to low educational attainment among African Americans and Hispanics, they are overrepresented in essential jobs, most of which are low-income jobs meaning they cannot exercise preventive measures such as social distancing, quarantine, or working from home [15]. African Americans and Hispanics have low education attainment limiting their opportunities for jobs. Consequently, they end up in jobs that expose them to COVID-19. The outcome is that African Americans and Hispanics have been disproportionately affected by COVID-19 socially, economically, and health-wise. Even before the pandemic, African Americans were already overrepresented among people living below the poverty line. With the pandemic and its accompanying economic disruption, more and more African Americans and Hispanics have been driven into poverty. Strict preventive measures such as quarantine, social distancing, and movement restrictions have exacerbated the already negative social-economic status of African Americans and Hispanics. African Americans increasingly found it difficult to get jobs in order to make ends meet. This means that even access to health care services is undermined for these minority populations.

The economic disruption of COVID-19 and its impact on minorities has had far-reaching implications for public health in the United States. African Americans and Hispanics already face challenges as far as health care access is concerned. They are overrepresented among the uninsured residents in the United States. With COVID-19, health care access for African Americans and Hispanics was further restricted. Bearing in mind that conditions such as diabetes and cardiovascular disease are prevalent among African Americans and Hispanics, the shrinking access to health care means that more people with chronic conditions increasingly find it difficult to get the health care services required [16]. The following section focuses on expounding the interactions of factors that have led to the disproportionate impact of COVID-19 among African Americans and Hispanics. More specifically, the section focuses on how low education attainment and misinformation combined to result in a disproportionate impact of COVID-19. Furthermore, how the selected five public health themes (evidence-based programs; public health leadership; communication and advocacy; vulnerable populations and health disparities; and effects of policy and evaluation) would address the health disparities issues will be expounded.

## Review

Epidemiological perspective of COVID-19 misinformation

Rationally, the prevalence of COVID-19 and misinformation will necessitate an efficient public health strategy that is similar to epidemiologic models to combat misinformation and myths. This may be accomplished by concentrating on the following areas: implementing evidence-based initiatives, enhancing surveillance, addressing health inequalities, and deploying public health specialists to combat infodemics. The epidemiological implications of misinformation were examined in the subsections below.

Drivers of Disparities

Early studies in the US showed that counties with high and diverse populations had higher COVID-19 infection rates, whereas those with less work mobility had low infection rates [7]. Additionally, due to the high percentage of people lacking formal education in the African American and Hispanic communities, misinformation is highly pervasive among these groups. This has significantly led to an increase in the dispersion of the virus among racial and ethnic groups but varies disproportionately. According to research conducted in the US, education, in addition to other factors like age, geographic location, and gender, is a significant factor that triggers the spread of false information. People with more education are significantly less likely to spread erroneous information [17,18]. Furthermore, populations with a high proportion of college-educated people had low infection rates compared to populations with low college-educated people [7]. This is because a college education is associated with high income and the ability to afford healthcare. Furthermore, the prevalence of co-morbid conditions increases with age. However, African Americans and Hispanics show the highest prevalence of co-morbid conditions. Therefore, due to the high prevalence of co-morbid conditions among African Americans and Hispanics, they possess an increased risk of adverse COVID-19 outcomes, including a high mortality rate [7].

Impact of Evidence-Based Public Health Approach on Misinformation

Despite structural efforts made as a result of the Evidence-based Public Health (EBPH) approach to increase access to vaccines and vaccination rates, African Americans are still significantly behind their counterparts in terms of vaccine uptake. Structural inequities, for instance, inadequate access to vaccines, have played a significant role in limiting the rates of vaccination among African Americans. African Americans have a greater mistrust in the healthcare system as a result of a long history of medical racism and discrimination, which includes unequal access to medical facilities, insurance, and treatment [19]. African Americans are in a situation where disinformation can flourish because of widespread suspicion and a lack of access to credible information regarding COVID-19 in the media and on social media platforms like Twitter [20,21]. WHO defines an infodemic as the spread of “false or misleading information in digital and physical environments during a disease outbreak" [22]. Infodemic may expose millions of individuals to false information that claims a disease to be a scam, that public health professionals are misrepresenting its severity or prevalence, and that vaccination may cause the condition, among others. The public may be less likely to take preventative action as a result of the infodemic, particularly among disadvantaged and uneducated populations [23]. During the peak of the COVID-19 pandemic, a lot of evidence was collected, but only a significant few policymakers and public health leaders used them accurately from the onset [24]. Escoffery et al. claim that an evidence-based approach is crucial for enhancing public health outcomes and lowering disparities and misinformation [25]. Similar to this, Brownson et al. [11] referred to EBPH as a tool for including the community and stakeholders in evaluation and decision-making and helping to disseminate high-quality outcomes. EBPH is a recommended approach to use in public health practice, and as a result, a necessary competency to support continuous improvement in public health programs by credible public health leaders [25,26].

There is a need to execute an (EBPH) strategy even if various methods to minimize inequities in COVID-19 distribution, such as effective vaccination distribution, reduction of mitigation, etc. have created considerable optimism. This is essential to lessen the unequal distribution of COVID-19 incidence and mortality rates across the country and to better prepare for upcoming pandemics. In order to apply the EBPH approach in public health settings and create a long-lasting model or framework that will work for each health concern, experts should be recruited, or future professionals should be trained. This involves improving community-level health education, ensuring that everyone has equitable access to medical facilities and services, effectively disseminating accurate data and statistics via reputable sources and media, making sure that mitigations are data-driven, and using other EBPH approaches [27,28].

Systemic Disadvantages and Social Determinants of Health

The COVID-19 race and ethnic disparities in the United States trace their origins to structural/systemic disadvantages. These disadvantages include low education levels, limited healthcare access, poverty, and the development of co-morbidities. These disadvantages set the stage for a disproportionate risk of negative COVID-19 outcomes [7]. African Americans and Hispanics historically had low college attainment compared to white non-Hispanic Americans [14,29]. Consequently, they are overrepresented among “essential” jobs in the transportation, food, and janitorial industries [8]. These jobs were exempted from restrictive measures set in place by the government to curb the surge of COVID-19. Furthermore, housing disadvantages mean African Americans live in densely populated neighborhoods with poor sanitary conditions making it difficult to practice social distancing measures. Furthermore, African Americans and Hispanics often utilize public transportation, further exposing them to COVID-19 infection.

Access to health is recognized under international human rights law as fundamental to fully realizing human rights [30]. Therefore, the right to health encompasses access to timely and appropriate health care as well as determinants of health. Determinants of health include racism, discrimination, residential segregation, poverty, and underlying medical conditions [30]. Structural and systemic racism, which entails interconnected institutions combine to perpetuate poverty, low-wage jobs, homelessness, and limited access to health care. As stated before, the type of work determines an individual’s level of risk for COVID-19 [30]. Data shows that over 40% of African Americans work in low-wage jobs which do not provide paid sick leaves, even increasing exposure to COVID-19 [30]. Similar to Dr. Kimberly Sue’s ethnography, segregation affects people’s access to social amenities such as water, healthy food, and sanitary services [31]. Segregation also enhances the risk of structural vulnerability; for example, exposure to environmental pollution is differentially distributed across geographic locations, leading to the development of diseases such as diabetes, kidney, and cardiovascular diseases and disproportionately affecting susceptible populations. African Americans and Hispanics disproportionately live in impoverished neighborhoods. These neighborhoods lack access to healthy food, clean water, hospitals, and other medical facilities. Consequently, these populations suffer from high rates of long-term health conditions. Furthermore, African Americans and Hispanics have historically been disparately diagnosed with chronic conditions, including hypertension, asthma, and diabetes. These underlying conditions make infection with COVID-19 more fatal among minority populations [30]. In fact, hypertension is among the highest risk factors for COVID-19 deaths among African Americans [30].

Therefore, the socioeconomic status of African Americans and Hispanics, which collectively include reliance on public transportation, poor living conditions in densely populated neighborhoods, overrepresentation in low-income jobs without sick leave, limited access to healthcare, and low education attainment, overexposes these populations to high COVID-19 infection.

Education attainment and misinformation

The impact of educational attainment has received little attention as a factor for the disproportionate distribution of COVID-19. Like other factors, low educational attainment among African Americans results from structural and systemic disadvantages [17]. Misinformation about COVID-19 has proliferated, posing a threat to public health in the United States. COVID-19 misinformation, including inaccurate health advice, how the virus spreads, and how it is cured, have contributed to COVID-19 spread in the country. A combination of technology and health literacy drove the proliferation of COVID-19 misinformation. Research has shown that fake social media accounts, bots, and trolls disguised as legitimate people are used to spread polarizing, unsolicited, and often fake/inaccurate information regarding COVID-19 to erode the trust of communities and populations, although this could not be ascertained at the beginning of the pandemic [32]. Often, misinformation targets uneducated populations used for political gain, and conspiracy agendas [33]. In fact, African Americans and Hispanics are more likely to be susceptible to misinformation due to low educational attainment among these populations.

Impact of COVID-19 misinformation

The propagation of misinformation has a far-reaching impact on the health of populations. Low education levels among African Americans and Hispanics are associated with limited health literacy [29]. Therefore, such populations are highly susceptible and skeptical of factual COVID-19 information, especially when disseminated by government authorities and health systems. This is due to deep distrust of the government establishment [34]. Furthermore, a combination of low education levels and low health literacy exposes African Americans to be susceptible to misinformation that places them at risk of contracting COVID-19. It also encourages these populations to engage in risky behavior. These risky behaviors include ignoring public health directives put in place to prevent transmission of COVID-19 such as social distancing and wearing masks, among others [35]. Essentially, COVID-19 misinformation undermines effective preventive measures such as social distancing and mask-wearing to the detriment of at-risk populations such as African Americans and Hispanics.

Due to limited educational attainment and thus limited health literacy among African Americans and Hispanics, there is an increased likelihood of vitalizing misinformation on social media platforms. The lack of sufficient health literacy and critical thinking makes certain populations susceptible to COVID-19 and misinformation, increasing their exposure to the virus. Misinformation contributes to vaccine hesitancy and refusal. Vaccine hesitancy entails taking too long to accept vaccination even when vaccines are readily available [35]. On the other hand, vaccine refusal entails intent not to get vaccinated. Vaccine refusal and hesitancy are fuelled by misinformation, which is fuelled by low education levels and health literacy. According to the US Surgeon General, misinformation is the one major threat facing vaccination efforts against COVID-19. The combination of low education levels and health literacy fuels susceptibility to COVID-19 misinformation among African Americans and Hispanics. This fuels vaccine hesitancy and reduces vaccine rates leading to preventable COVID-19 deaths, especially among African Americans and Hispanic populations. 

A study of COVID-19 vaccine hesitancy in the US found that 41.6% of African Americans and 30.2% of Hispanics were unwilling to vaccinate [36]. Factors driving low vaccine rates among African Americans and Hispanics, especially lower education attainment, existed among these populations even before the pandemic. Notably, studies have shown that education attainment influences susceptibility to misinformation [37-39]. Higher education attainment is associated with analytical thinking, critical thinking, numeracy skills, and reflective thinking, which are critical in processing misinformation [37]. Thus, lower levels of education are associated with a higher vulnerability to misinformation.

Recommendations

**Figure 1:**
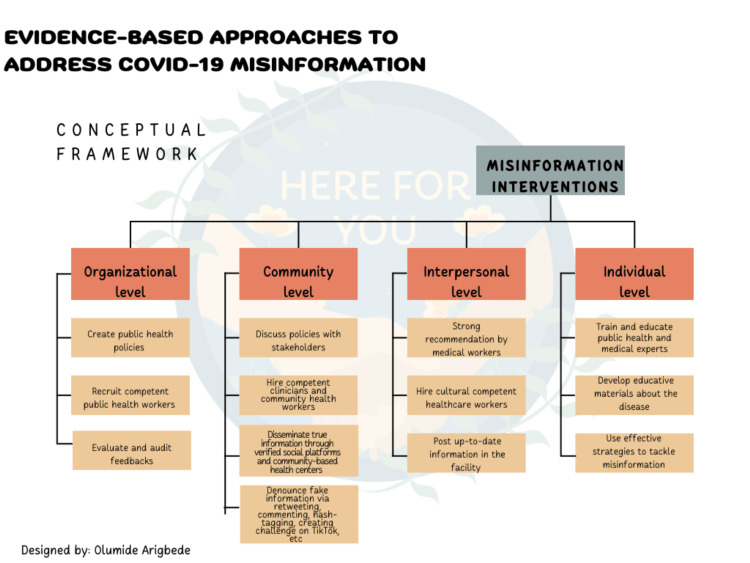
Conceptual Framework for Tackling COVID-19 Misinformation Image by Olumide M. Arigbede

In order to address the disparate distribution of COVID-19 among African Americans, the focus should be placed on addressing factors that contribute to increased risk of exposure and high mortality. These factors are directly linked to socioeconomic disadvantages that face African Americans and Hispanics. From low-income jobs that increase exposure to living conditions that limit preventive measures and limited access to health care such as insurance must be addressed. More specifically, the focus should be on enhancing health literacy to tackle COVID-19 misinformation among these minority populations. It is clear that a combination of low educational attainment is prevalent among African Americans. Consequently, health literacy is limited, increasing vulnerability to misinformation. Misinformation, in turn, increases risky behavior and promotes vaccine hesitancy and refusal. In the short-term, vaccine hesitancy should be acknowledged and addressed by countering misinformation that is fueling it. To significantly reduce this trend and capitalize on the capability of leveraging several media types to convey accurate and validated information, interventions from a variety of stakeholders are needed. This calls for robust health literacy especially targeting COVID-19-related misinformation at the community level. Open communication between health care stakeholders and minority populations is essential in countering COVID-19 misinformation. Additionally, practical, and tangible initiatives must address the mistrust of institutions stemming from historical injustices. This is because experiences of injustice against minorities exacerbate their vulnerability to misinformation. At the health care level, healthcare providers should build relationships and recommend vaccines to African American and Hispanic patients in every encounter. This is also an opportunity to address COVID-19 concerns, including vaccine safety, and coronavirus transmission, among others. Lastly, developing competent, multimodal, and culturally competent communication strategies and the application of efficient evidence-based strategies (Figure 1) across minority communities is important in countering misinformation. Communication should focus on fact-finding, myth dispelling, rumor control, and message monitoring. Due to low educational attainment and limited health literacy, it is important to constantly monitor and adjust communication at the community level.

Summary of the Conceptual Framework

The issue of misinformation, also known as infodemic, has a significant influence on society, particularly with the devastating COVID-19 and among the historically marginalized people in the US. According to the conceptual framework, this menace of false information may be combated on four different levels: individual, interpersonal, community, and organizational. To successfully convey and provide accurate information about a disease, medical and public health workers must be well-trained at the individual level. As a result, people will be more able to filter information concerning diseases and become less vulnerable to them. While the community must develop policies on how to convey the required information to the population and be prepared to reject false information through all available media, interpersonal levels must be strong to increase the public's trust in public health/medical practitioners. To eradicate false information about COVID-19 or other diseases, organizations must develop public health policies, hire competent health personnel, and evaluate feedback.

## Conclusions

Several factors combine to increase the risk for COVID-19 infection and adverse outcomes among African Americans and Hispanics. Notably, structural, and systemic disadvantages facing African Americans predispose them to a higher risk of COVID-19. Notably, low socioeconomic status and negative health determinants place these populations at increased risk of COVID-19. Low education attainment among these populations means they occupy low-wage jobs without paid sick leave in essential industries. Consequently, their COVID-19 exposure risk is in comparison to populations in other high-income jobs. Education also affects health literacy and thus vulnerability to misinformation. African Americans and Hispanics are victims and, by extension, vulnerable to COVID-19 misinformation disseminated for political gains. Low educational attainment among African Americans and Hispanics undermines their ability to synthesize information to sieve facts regarding COVID-19. The outcome is that vulnerability to misinformation encourages these populations to engage in risky behavior. For instance, susceptibility to COVID-19 misinformation increases vaccine refusal and hesitancy, which leads to preventable deaths. Moreover, susceptibility to COVID-19 misinformation encourages these minority populations to disregard effective preventive measures such as social distancing and wearing face masks.
